# Dissociable Catecholaminergic Modulation of Visual Attention: Differential Effects of Catechol-O-Methyltransferase and Dopamine Beta-Hydroxylase Genes on Visual Attention

**DOI:** 10.1016/j.neuroscience.2019.05.068

**Published:** 2019-08-01

**Authors:** Nir Shalev, Signe Vangkilde, Matt J. Neville, Elizabeth M. Tunbridge, Anna C. Nobre, Magdalena Chechlacz

**Affiliations:** a Department of Experimental Psychology, University of Oxford, Oxford, UK; b Oxford Centre for Human Brain Activity, University of Oxford, Oxford, UK; c Wellcome Centre for Integrative Neuroimaging, University of Oxford, Oxford, UK; d Department of Psychology, Center for Visual Cognition, University of Copenhagen, Copenhagen, Denmark; e Oxford NIHR Biomedical Research Centre, University of Oxford, Churchill Hospital, Oxford, UK; f Oxford Centre for Diabetes, Endocrinology and Metabolism, Radcliffe Department of Medicine, University of Oxford, Oxford, UK; g Department of Psychiatry, University of Oxford, Oxford, UK; h Oxford Health NHS Foundation Trust, Oxford, UK; i Centre for Human Brain Health, University of Birmingham, Birmingham, UK; j School of Psychology, University of Birmingham, Birmingham, UK; k Beckman Institute for Advanced Science and Technology, University of Illinois at Urbana-Champaign, USA

**Keywords:** attentional selection, sustained attention, COMT, DBH, individual differences, catecholamines

## Abstract

Visual attention enables us to prioritise behaviourally relevant visual information while ignoring distraction. The neural networks supporting attention are modulated by two catecholamines, dopamine and noradrenaline. The current study investigated the effects of single nucleotide polymorphisms in two catecholaminergic genes – COMT (Val^158^Met) and DBH (444 G/A) – on individual differences in attention functions. Participants (*n* = 125) were recruited from the Oxford Biobank by genotype-based recall. They were tested on a continuous performance task (sustained attention), a Go/No-Go task (response inhibition), and a task assessing attentional selection in accordance with the Theory of Visual Attention (TVA). We found a significant effect of DBH genotype status on the capacity to maintain attention over time (sustained attention) as measured by the continuous performance task. Furthermore, we demonstrated a significant association between COMT genotype status and effective threshold of visual perception in attentional selection as estimated based on the TVA task performance. No other group differences in attention function were found with respect to the studied genotypes. Overall, our findings provide novel experimental evidence that: (i) dopaminergic and noradrenergic genotypes have dissociable effects on visual attention; (ii) either insufficient or excessive catecholaminergic activity may have equally detrimental effects on sustained attention.

## Highlights

• Catecholaminergic genotypes have dissociative cognitive effects on visual attention.
• DBH (444 G/A) polymorphism affects sustained attention.
• COMT Val^158^Met polymorphism affects perceptual threshold in visual attention.
• Both too little and too much catecholamines may detrimentally impact sustained attention.

## Introduction

The term ‘visual attention’ refers to the set of cognitive processes that enables an individual to stay focussed on the task at hand by selecting behaviourally relevant visual information while ignoring distractors (Posner, 1980, Nobre and Kastner, 2014). Attention-related mechanisms are often distinguished on the basis of how attention is focussed or distributed over time (‘sustained attention’ and ‘alertness’; e.g., Parasuraman, 1998, Petersen and Posner, 2012, Posner and Petersen, 1990, Robertson et al., 1997, Sturm and Willmes, 2001) or over space or objects (i.e., ‘orienting attention’; Nobre and Mesulam, 2014, Petersen and Posner, 2012, Posner and Petersen, 1990). Most theories of attention share the basic assumption that it is best understood as a heterogeneous, multifaceted system, and different theoretical frameworks describe distinct parameters considered essential to specific types of attention (e.g., Duncan and Humphreys, 1989, Posner and Petersen, 1990, Desimone and Duncan, 1995, Parasuraman, 1998). For example, a prominent approach to understanding visual attention is to emphasise the processes governing the attentional selection (e.g., Desimone and Duncan, 1995), and describe the multiple factors underlying its efficiency (e.g., Bundesen, 1990).

A growing body of evidence suggests that distinct mechanisms of attention are differentially modulated by multiple neurotransmitters, including two catecholamines, dopamine and noradrenaline (for review see Posner, 2008, Marrocco and Davidson, 1998, Thiele and Bellgrove, 2018). The main evidence supporting the catecholaminergic influences on attention comes from pharmacological studies, conducted in both animals and humans (e.g., Clark et al., 1989, Ward and Brown, 1996, Coull, 1998, Coull, 2001, Wise, 2004). Recently, the roles of dopamine and noradrenaline in attention have been also investigated by means of human studies examining the association between functional single-nucleotide polymorphisms (SNPs) and cognitive performance as well as different phenotypes symptomatic of attention disorders (e.g., Daly et al., 1999, Roman et al., 2002, Hawi et al., 2003, Bellgrove et al., 2005a, Bellgrove et al., 2005b, Bellgrove and Mattingley, 2008, Bellgrove et al., 2009). Specifically, it has been shown that functional polymorphisms in several dopaminergic and noradrenergic genes are associated with asymmetries of spatial attention, including lateralized target detection and distractor suppression (e.g., dopamine transporter gene DAT1, Bellgrove et al., 2007, Greene et al., 2010, Newman et al., 2014, Newman et al., 2012, Zozulinsky et al., 2014; dopamine D2 receptor, Zozulinsky et al., 2014), top-down control of attention (e.g., catechol-O-methyltransferase COMT and DAT1, Schneider et al., 2015), distractibility (e.g., DAT1, Holmboe et al., 2010), and sustained attention (e.g., dopamine beta-hydroxylase DBH, Greene et al., 2009).

The major sources of catecholamines in the human brain are subcortically located neurons, with noradrenergic pathways originating in the locus coeruleus, and dopaminergic pathways originating in the ventral tegmental area and the substantia nigra (Aston-Jones and Cohen, 2005, Sara, 2009). While catecholamine-secreting neurons project throughout the cortex, the prefrontal cortex (PFC) projections are commonly linked to various aspects of attention, in particular inhibitory control, sustained attention, and selective attention (Briand et al., 2007, Noudoost and Moore, 2011, Chandler et al., 2014, Clark and Noudoost, 2014, Ranganath and Jacob, 2016). Despite overlapping PFC projections, dopamine and noradrenaline differ in their specific neuronal targets and their influences on behavioural functions, including the cognitive domains orchestrated by the PFC (for review see Chandler et al., 2014). Thus, understanding how dopaminergic and noradrenergic modulation of the PFC differentially influences attention-related functions may provide insight into how distinct facets of attention are controlled and facilitate the pharmacological targeting of specific attention deficits in brain disorders (Ernst et al., 1998, Engert and Pruessner, 2008, Del Campo et al., 2011, Gamo and Arnsten, 2011).

The current study aimed to explore common and dissociable effects of catecholamines on visual attention by examining the impact of single nucleotide polymorphisms in two catecholaminergic genes – COMT (Val^158^Met) and DBH (444 G/A) – on inter-individual variability in performance on tasks measuring different aspects of attention function. In contrast to previous studies, we systematically explored the influence of catecholaminergic genotypes on distinct attention-related functions rather than restricting the enquiry to a single attentional function. We employed three theoretically motivated tasks, focussing on either the mechanisms of how attention is focussed and distributed over time or factors underlying efficiency of attentional selection. Specifically, we used two versions of the modified Continuous Performance Task (CPT), differing in the target–distractor ratio in order to measure either sustained attention or response inhibition (Shalev et al., 2016, Shalev et al., 2018b, Young et al., 2018). Compared to other frequently used measures of sustained attention and response inhibition (e.g., Robertson et al., 1997, Ballard, 2001), the employed here CPT tasks use masking and conjunctive sets of stimuli. These modifications have been previously shown to increase sensitivity to temporal changes in performance patterns (Shalev et al., 2016, Shalev et al., 2018b). To test attentional selection, we used a task implementing the computational theory of visual attention (TVA, Bundesen, 1990). The TVA model assesses attentional selection and capacity based on assumptions of limited processing resources and of the biased competition model of attention (Desimone and Duncan, 1995). According to TVA, visual attention is described as a parallel processing race in which visual objects compete simultaneously for representation in a short-term memory store with a limited capacity. The winners of the race are encoded in the short-term memory store and made available for conscious recognition and action. The probability of winning the race and the processing rate of a given object are influenced by attentional weights (i.e., the processing capacity allocated to each object) and perceptual biases (i.e., the tendency to categorise the object as belonging to a certain category). The mathematical implementation of the TVA model enables estimation of five theoretical parameters: visual short-term memory capacity, processing speed, perceptual threshold, spatial bias and top-down selectivity index (see methods section for full details; Bundesen, 1990, Kyllingsbaek, 2006; Kyllingsbaek et al., 2011). Importantly, it has been previously shown that attentional selectivity and capacity measures derived in accordance with the TVA model are unrelated to sustained attention measures (McAvinue et al., 2012, Shalev et al., 2018a). This ensures that the tasks employed here indeed provide measures of distinct, independent attention functions.

The Val^158^Met COMT polymorphism (rs4680; guanine to adenine missense mutation at position 158, resulting in valine [Val] to methionine [Met] substitution; Chen et al., 2004, Lachman et al., 1996, Tunbridge et al., 2006, Tunbridge et al., 2007) affects activity of the COMT enzyme, involved in the degradation of the cortical dopamine (Matsumoto et al., 2003a, Matsumoto et al., 2003b, Tunbridge et al., 2006, Yavich et al., 2007). The Met allele produces a COMT isoform with reduced enzymatic activity, and subsequently Met/Met carriers are predicted to have the highest amounts of prefrontal dopamine, and Val/Val carriers the lowest (Lotta et al., 1995, Chen et al., 2004, Tunbridge et al., 2006, Tunbridge et al., 2007). The second studied here SNP, the 444 G/A DBH polymorphism (rs1108580; guanine to adenine substitution at position 444 within exon 2 of the DBH gene located on the chromosome 9) affects activity of DβH enzyme, which influences the balance between the two catecholamines by converting dopamine to noradrenaline (Kaufman and Friedman, 1965, Bourdelat-Parks et al., 2005, Zhang et al., 2010). The A allele has been associated with lover levels of the DβH enzyme and thus less noradrenaline compared to the G allele (Cubells et al., 1998, Cubells et al., 2000, Cubells and Zabetian, 2004). Both COMT (Val^158^Met) and DBH (444 G/A) have been previously linked to variability in cognitive performance. The Val^158^Met COMT polymorphism has been predominantly studied with respect to its impact on executive function and working memory (e.g., Egan et al., 2001, Tunbridge et al., 2004, Bertolino et al., 2006, Barnett et al., 2007, Frank et al., 2007, Farrell et al., 2012, Tunbridge et al., 2012, Jaspar et al., 2015). Although, some studies have also examined its effects on attention-related functions, in particular cognitive benefits of higher dopamine levels on spatial orientation bias i.e., tendency to orient attention towards one hemi-field (Zozulinsky et al., 2014), attentional selection (top-down control; Schneider et al., 2015), and distractibility (Holmboe et al., 2010). While prior studies have only demonstrated a strong effect of 444 G/A DBH polymorphism on spatial working memory performance (Parasuraman et al., 2005, Greenwood et al., 2009), another DBH polymorphism (rs1611115, − 1021 C/T SNP; cysteine to thymidine substitution at position 1021 within the promoter region of the DBH gene) has been reported to affect sustained attention (Greene et al., 2009).

Taken together, we hypothesised that we would find differential effects of COMT (Val^158^Met) and DBH (444 G/A) polymorphisms on visual attention. Specifically, we predicted that while the COMT Val^158^Met genotype status would affect attentional selection measures, namely visual short-term memory capacity, spatial bias and top-down selectivity index estimated in accordance with the TVA model, the DBH 444 G/A genotype status would be associated with differences in sustained-attention capacity.

## Experimental Procedures

### Participants

One hundred and twenty-five participants (mean ± SD age = 44.8. ± 4.43) were recruited from the Oxford Biobank by genotype-based recall to warrant comparable allele frequency. The Oxford Biobank consists of a sample of more than 7124 genotyped male and female individuals (age 30–50) in Oxfordshire who have consented to be re-approached for a ‘recruit-by-genotype’ participation in various biomedical studies. Given known sexual dimorphisms in the function of one of the studied genes (COMT, Tunbridge and Harrison, 2011), only male participants who were either homozygous for COMT Val^158^Met or DBH 444 G/A were selected and contacted by post (*n* = 706). All individuals who responded positively (*n* = 174) were invited to attend cognitive testing as described below. Subsequently, a total of 125 participants completed one testing session conducted in a double-blind fashion (genotype status unknown to both experimenter and participant at the time of testing). Two separate sets of analyses were conducted (see Statistical Analyses section), and thus participants were split into groups based on either DBH (A/A group, *n* = 42; mean age 44.6; SD 4.6; G/G group, *n* = 47; mean age 44.9; SD 4.2; G/A group; *n* = 36; mean age 45; SD 4.4) or COMT (Val/Val group, *n* = 42; mean age 45.9; SD 4.2; Met/Met group, *n* = 43; mean 45.1 age; SD 4.3; Val/Met group, *n* = 40; mean age 43.4; SD 4.3) genotype status. All participants had either normal or corrected-to-normal vision. Both left- and right-handed participants were recruited for the study, and the hand dominance was assessed according to the Edinburgh handedness inventory (Oldfield, 1971; mean score 61.9, SD 51; 101 right handed, 13 left handed, 11 ambidextrous). None of the genotype groups differed significantly in their mean age and handedness. All study participants provided written informed consent, in compliance with the relevant protocols approved by the University of Oxford Central University Research Ethics Committee. All experimental procedures were conducted in accordance with the latest version of the Declaration of Helsinki.

### General procedure

All participants were tested on three tasks, in the following order: 1) a Continuous Performance Task (CPT) to measure sustained attention (CPT-SA; approximately 15 min), 2) a CombiTVA task to assess selective attention (TVA-based assessment; approximately 45 min), and 3) a CPT Go/No-go version (CPT-GNG; approximately 15 min) to measure response inhibition. The whole testing session lasted 1.5 h. A personal computer with Intel i7 processor and a dedicated 2GB AMD video card was used for displaying stimuli and recording data. The two CPT tasks were generated and administered using Presentation software (Neurobehavioral Systems, Albany, CA), and the CombiTVA paradigm was generated and administered using E-prime 2 Professional software (Psychology Software Tools, Inc.). The stimuli were presented on a ViewSonic V3D245 LED monitor, with screen resolution of 1080 × 1920 and a screen refresh rate set at 100 Hz allowing display times varied in 10-ms gaps. At the start of each task all stimuli were preloaded to memory to minimise temporal noise.

### CPT-SA and CPT-GNG tasks

We used two versions of the Continuous Performance Task (CPT; Shalev et al., 2018b), differing only in the target–distractor ratio as described below. Following the convention in testing inhibition and sustained attention, the CPT version with the high proportion of targets was used to measure response inhibition as a ‘Go/No-Go’ task i.e., CPT-GNG task (e.g., Young et al., 2018) and the version with low target proportion was used to test sustained attention i.e., CPT-SA task (e.g., Shalev et al., 2016, Shalev et al., 2018b).

### Design and stimuli

**Fig. 1:**
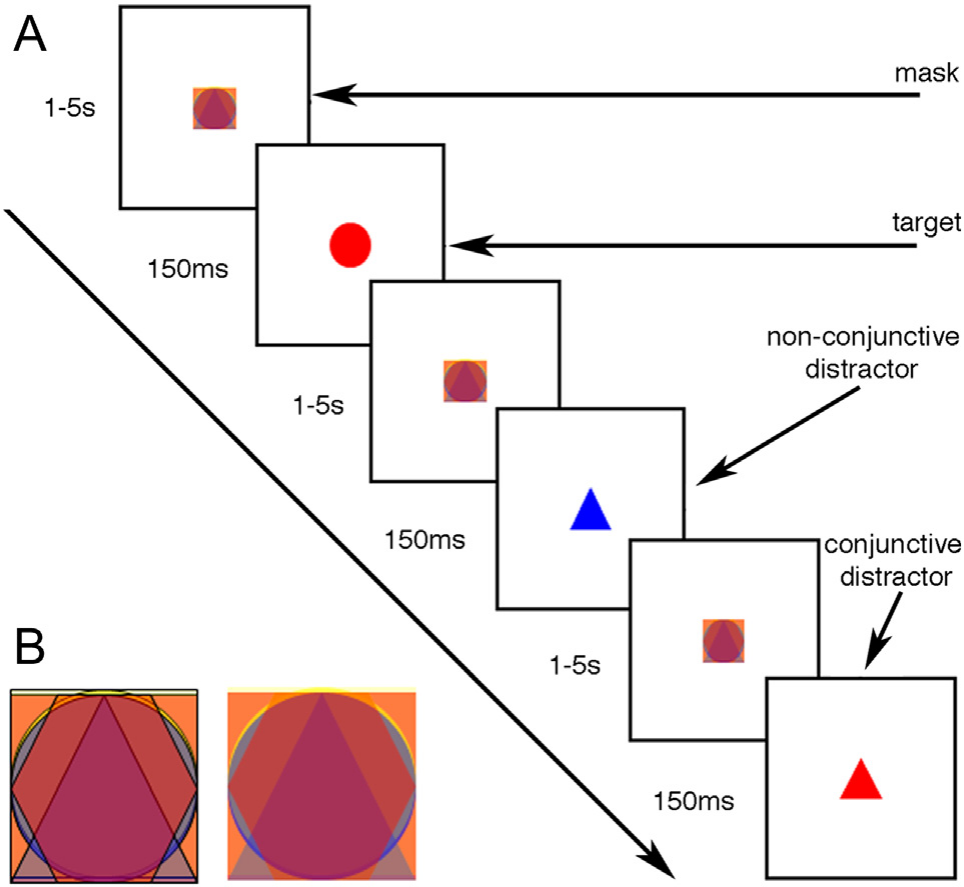
**Schematic of the CPT-SA and CPT-GNG tasks: (A)** the basic outline of the CPT task (see methods section for full details); **(B)** the two alternating mask-images.

A coloured mask (Mask), comprising of four superimposed figures in different colours (square, triangle, circle and hexagon) appeared at the centre of the screen. The total size of the mask occupied 3° × 3° visual angle. In order to avoid habituation effects, we generated minor movement of the Mask. The movement was generated by alternating every 10–20 ms between two mask-images, one of which had thicker outlines for the superimposed figures (the two alternating mask images are illustrated in Fig. 1B). The mask appeared at the centre of the screen and disappeared only when it was replaced by either a target or a distractor shape for 150 ms; the mask then reappeared immediately, generating pre- and post-masking of each target or distractor. The target shape was a red circle, and the distractor stimuli were either similar in colour to the target (red hexagon and red triangle), similar in shape (blue circle and yellow circle), or completely different (yellow hexagon and blue hexagon). All distractor types appeared in equal proportion. All distractors and target shapes fit in a square of 3° × 3° visual angle. The inter-stimulus interval was jittered between 1000 and 5000 ms (See Fig. 1A for a schematic outline of the experimental procedure). Participants were told that the constant shape which appeared at the centre of the screen (the Mask) would be replaced briefly by another shape every few seconds. The task was to press the response button as quickly as possible whenever participants recognised a *red circle* at the centre of the screen. Participants were further instructed to do nothing when they saw any other shape.

### Procedure

The task started with a short practice block (15 trials), and the experimenter monitored participants' responses at this stage to ensure the instructions were clear. After finishing the practice session, the participants performed the whole session without any break until the task terminated after approximately 10 min. The task comprised 180 trials. In the CPT-SA task, the target appeared on 60 trials (33% *target)*, and there were 120 *distractor* trials (67%) in which one of six possible distractors appeared on the screen with equal probability and in randomised order (red square / red triangle / blue circle / blue triangle / yellow circle / yellow triangle). The CPT-GNG design was the same except for the larger proportion of target stimuli (67%) relative to distractor stimuli (33%).

### Estimation of the CPT parameters

For each participant, we extracted the following behavioural data: the number of correct responses to targets and their associated reaction times, the number of correct rejections of distractors, the number of false alarms (i.e., classifying a distractor as a target), and the number of missed targets. The time window for response was set to 1 s. To assess sustained attention, we calculated two outcome measures based on the CPT-SA task performance: the standard deviation of reaction times during the entire task (RT-SD) representing the stability of responses throughout the task; and the percentage change in perceptual sensitivity between two task-halves (d’-change), representing the capacity to maintain attention over time on task. The d’ was calculated based on the Signal Detection Theory (SDT) as the distance between the standardised values of correct response and false alarm rates (Green and Swets, 1966, Stanislaw and Todorov, 1999). To assess response inhibition based on the CPT-GNG task performance, we extracted the number of false-alarms and calculated the percentage of responses classifying a distractor as a target (false alarms). For the purpose of supplementary analysis, we also calculated perceptual sensitivity d’ based on the CPT-GNG task performance. For a detailed discussion on the continuous performance task design and the validity of derived sustained attention and response measures see Shalev et al. (2018b).

### CombiTVA task

**Fig. 2:**
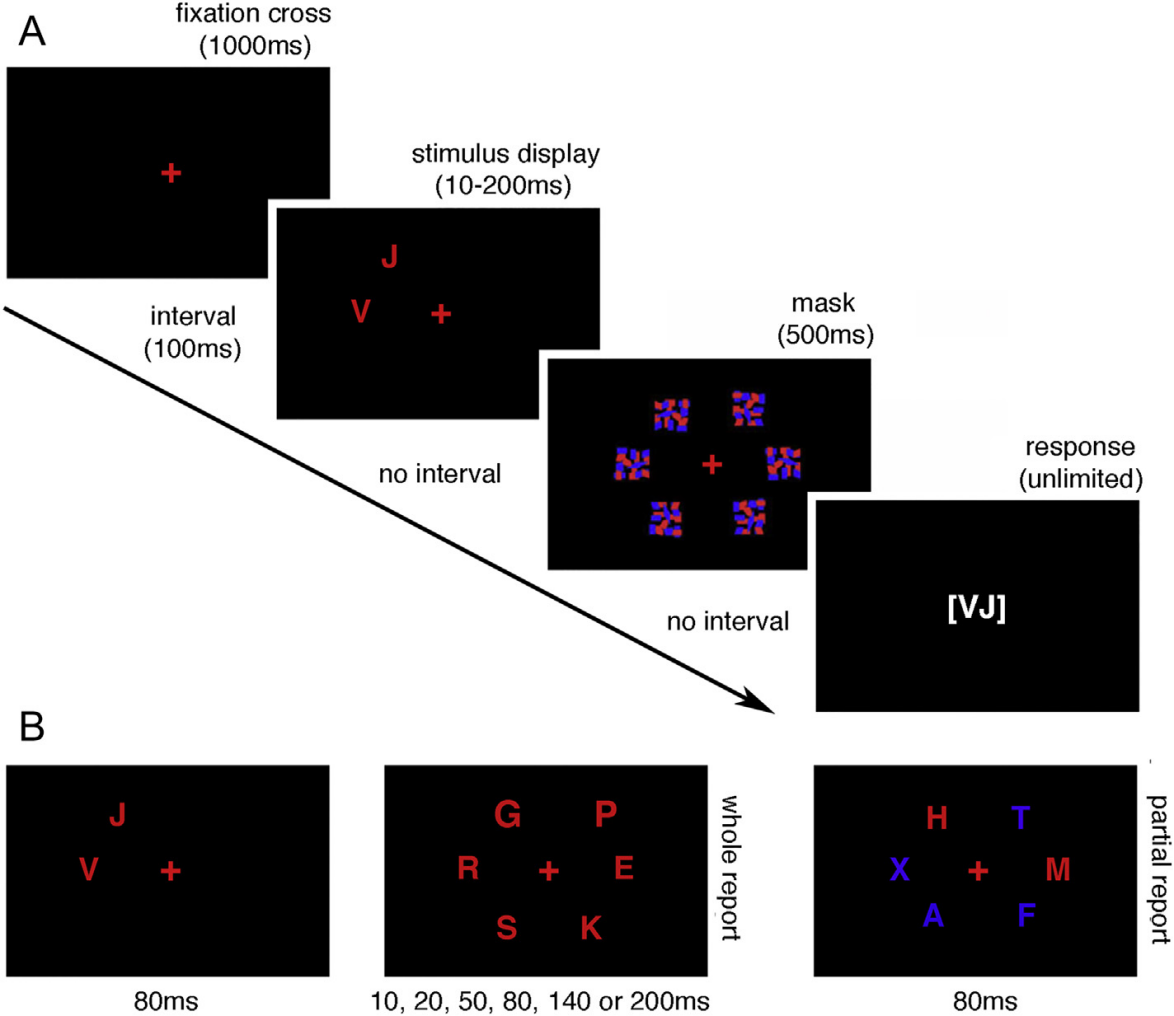
**Schematic of the CombiTVA task: (A)** the basic outline of an experimental trial (the full task consisted of nine blocks of 36 trials); **(B)** types of stimulus displays and exposure times used in the whole and partial report arrays (see methods section for full details).

We employed the CombiTVA paradigm (Vangkilde et al., 2011; Fig. 2) to assess attentional selection parameters based on Bundesen's TVA framework. Both whole- and partial-report tasks were intermixed on different trials (Bundesen, 1990).

### Design and stimuli

At the beginning of each trial, a red fixation cross appeared in the centre of the screen for 1000 ms, followed by a blank screen presented for 100 ms and then by the stimulus display. The stimulus display could be of one of two conditions, presented in random order. In *whole-report* arrays either two or six red letters appeared on the screen; in *partial-report* arrays two red target letters and four distractor blue letters appeared on the screen. The letters were presented within six fixed placeholders equally distributed on the perimeter of an invisible circle (r = 7.5° of visual angle). The stimulus display consisted of letters chosen randomly without repeats from a set of 20 capital letters (ABDEFGHJKLMNOPRSTVXZ) with Arial font size corresponding to 2.7° × 2.3° of visual angle. The display appeared for one of six fixed durations of 10, 20, 50, 80, 140 or 200 ms (randomly presented and equally distributed) and was followed by a masking noise on each of the fixed placeholders lasting 500 ms. Following the mask, participants were presented with a blank response display and were prompted to recall as many red letters as they could, using the computer keyboard, and to press ‘SPACE’ key when done. The response display appeared for an unlimited time and the reported letters were visible on the screen until the initiation of the next trial following the press of ‘SPACE’ key.

### Procedure

The task started with a short practice block (24 trials), during which the experimenter monitored participants' responses to ensure that they understood the task instructions. Following the practice session, participants performed nine experimental blocks consisting of 36 trials each. The six possible exposure times of the stimulus displays, as well as the different three types of stimulus display appeared in a random order throughout the task. The target and distractor letters were chosen randomly on each trial. The participants were told that their reaction speed was not being monitored, and they should report all the red letters they were “fairly certain” of having seen and refrain from pure guessing. Such instructions are commonly used in TVA based tasks (e.g., Vangkilde et al., 2011, Vangkilde et al., 2012, Habekost et al., 2014). Following practise block and then each experimental block, the participants were informed of their accuracy rate. They were asked to try to maintain an accuracy range of 80%–90%; they were told that if their accuracy was higher, they should try to be less conservative when reporting letters, conversely, if their accuracy was lower, it meant they were guessing too many letters and they should try to be more accurate (more conservative). The whole procedure lasted approximately 45 min.

### Estimation of TVA parameters

The TVA model (Bundesen, 1990) is a mathematical formalisation based on the “biased competition” account of visual attention. Visual categorizations of individual items (i.e., ascribing features to objects) are proposed to compete to be encoded into a limited capacity visual short-term memory (VSTM). The categorisation of a visual element is accomplished once it has been encoded to VSTM. This race model is normally described by two main equations: the rate equation and the weight equation. The rate equation describes the rate *v*(*x*, *i*) at which a particular visual categorisation ‘*x* belongs to *i*’ is encoded into VSTM. The rate is determined as a product of three terms: *η*(*x*, *i*) which represents the strength of the sensory evidence in favour of categorising *x* as belonging to category *i*; *β*_*i*_ which represents the perceptual decision bias associated with category *i*; and $\frac{W_{x}}{\sum_{z\in S}W_{z}}$ which determines the relative attentional weight of object *x* divided by the sum of the attentional weights of all objects within the visual field (S). These three terms comprise the rate equation:

$$v\left(x,i\right)=\eta \left(x,i\right)\beta_{i}\left\{\frac{W_{x}}{\sum_{z\in S}W_{z}}\right\}$$

The sum of all rate values (*v*) across the visual field defines the overall processing speed (*C*), formally:

$$C=\sum_{x\in S}v\left(x\right)=\sum_{x\in S}\sum_{i\in R}v\left(x,i\right)$$

The second key equation, the weight equation, describes how attentional weights (*w*-values) are allocated to the perceived elements according to their pertinence value *π*_*j*_. The pertinence value is defined by the momentary importance of attending a perceived element *x* as belonging to a category *j*, where *R* is the set of all categories *η*(*x*, *j*). The weight equation:

$$W_{x}=\sum_{j\in R}\eta \left(x,j\right)\pi_{j}.$$

Finally, we used a partial-report paradigm where participants were requested to attend and report targets while ignoring irrelevant distractors (defined by a colour feature). Under the assumption that every target on a given display has approximately the same weight (*w*_*target*_), and every distractor has the same weight (*w*_*distractor*_) we determine the α value which defines the efficiency of top-down control as

$$\alpha =\frac{W_{\mathrm{distractor}}}{W_{\mathrm{target}}}$$

When applied to the CombiTVA data, these equations (see also, Bundesen, 1990) allow for the extraction of multiple independent theoretical parameters representing different aspects of attention (Vangkilde et al., 2011). The extraction of the theoretical attentional parameters from TVA-based data is based on a maximum-likelihood fitting procedure introduced by Kyllingsbaek (2006) and elaborated by Dyrholm et al. (2011). The output of the fitting algorithm includes five theoretical parameters: (1) Parameter *K* is an estimation of the visual short-term memory capacity, measured in number of letters that can be stored; (2) Parameter *t*_0_ is the perceptual threshold, defined as the minimum exposure duration required to evoke conscious perception, measured in milliseconds; (3) Parameter *C* is the visual processing speed, or processing rate, measured in number of letters processed per second; (4) The spatial bias parameter *w*_*index*_ which represents the ratio between the sum of the attentional weights assigned to items on the left, and the overall sum of all attentional weights. The parameter ranges between 0 and 1, with a value of 0.5 indicating symmetrical attentional weighting; a value closer to 0 indicates an attentional bias to the right, and a value higher than 0.5 indicates an attentional bias to the left side of the visual field; (5) The top-down selectivity index α defined as the ratio between the attentional weights allocated to a distractor and to a target. The resulting α value ranges between 0 and 1, with the lowest score indicating perfect selectivity (no attentional weight given to irrelevant distractors). In total, the applied model had 9 degrees of freedom (dfs): *K*, 5 dfs (the *K* value reported is the expected *K* given a particular distribution of the probability that on a given trial *K* = 1, 2, 3, 4, 5, and 6); *t*_0_, 1 df; *C*, 1 df; *w*_*index*_, 1 df; and α, 1 df. For a detailed overview of the TVA-derived attentional selection parameters and their correlates, see Habekost (2015).

The equations are implemented in the MATLAB (MathWorks Inc.) software, the LibTVA modelling toolbox (Dyrholm, 2011) which is available from the website http://www.machlea.com/mads/libtva.html.

The supplementary analyses examining how COMT genotype status affects the perceptual threshold were carried out using raw data instead of *t*_0_ parameter calculated as described above. Specifically, based on the raw data we estimated and entered into our analyses, the mean number of errors made and the mean number of identified letters at each exposure duration (10, 20, 50, 80, 140 and 200 ms).

### Model diagnostics

**Fig. 3:**
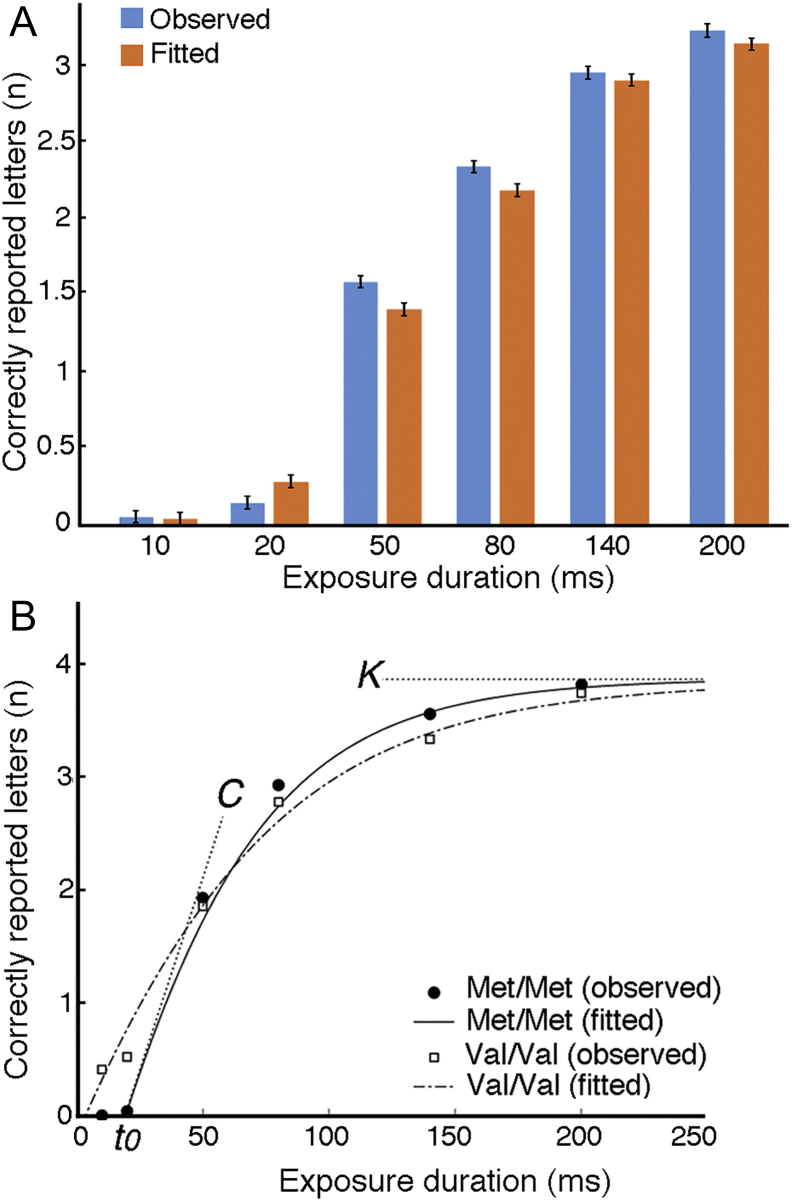
**TVA model diagnostics. (A)** Comparison between the observed data (mean number of correctly reported letters) and the data as predicted by the fitted TVA model. **(B)** Example of TVA model performance in two participants, one with Met/Met and one Val/Val genotype, illustrating the relation between observed and fitted mean scores. The observed data are plotted as function of exposure duration and the curves represent TVA-based fits (maximum-likelihood fits). The graph shows three TVA parameters: K (the visual short-term memory capacity), t_0_ (the perceptual threshold), and C (the processing speed).

We performed the goodness-of-fit calculations to provide estimation of the variation in the observed individual mean scores accounted for by the maximum-likelihood fits and the difference between the observed and the predicted data. The variation was calculated per participant as the R^2^ between the observed and predicted values. The difference between the observed and the predicted mean scores was calculated per participant as the squared numerical difference between the observed and the predicted scores for each condition divided by the number of conditions and then taking the root of the result. The obtained maximum-likelihood fits were excellent, accounting for an average of 88% of the variation in the observed individual mean scores (i.e., the percentage of variance in the observed individual mean scores accounted for by the maximum-likelihood fits), and correspondingly, the difference between observed and predicted mean scores was on average of 0.26 across all conditions. Please see also Fig. 3 for a direct comparison between the observed (i.e., mean number of correctly reported letters) and the predicted data at different exposure durations. The mean Pearson's correlation between the observed data and the predicted data was r = 0.94 (p ≪ .001). Finally, we conducted ANOVA tests for independent samples, with the variation in the observed individual mean scores and the difference between the observed and the predicted data as the dependant variables, and the genotype groups (COMT and DBH) as the independent variables. The ANOVA tests indicated that there were no differences between genotype groups in the ability of the model to explain the observed performance (all p's ≫ 0.1).

### Statistical analyses

To assess the effect of DBH 444 G/A and COMT Val^158^Met genotype status on different aspects of attention, we used a regression analysis with the two genotypes being used as predictors, and task indices as the dependent variables. We employed a stepwise fitting procedure, in which the first step included the genotype status, and the second step included the interaction between the two genotypes. In accordance with our hypotheses as presented in the introduction, we expected to find a differential effect of the genotype status (DBH 444 G/A versus COMT Val^158^Met genotype) on distinct measures of attention as assessed by the three tasks. The dependent variables were: 1) d’-change, representing capacity to maintain attention over time (sustained attention) based on CPT-SA task performance; 2) RT-SD, representing response stability as assessed based on CPT-SA task performance; 3) false-alarms rate, representing response inhibition as assessed based on CPT-GNG task performance; further dependent variables estimated based on the CombiTVA task performance included: 4) Visual-Sort Term Memory (VSTM) capacity; 5) Perceptual threshold; 6) Visual processing speed; 7) Top-down selectivity index; and 8) Spatial bias.

Following the regression analysis, we conducted a series of additional analyses to further explore the observed effects. To do so, we carried out a series of t tests and ANOVAs with either DBH or COMT Val allele dosage as the independent factors.

All statistical analyses were performed using either MATLAB (MathWorks Inc.) or SPSS (Ver 24; IBM Corp, 2016).

## Results

**Fig. 4:**
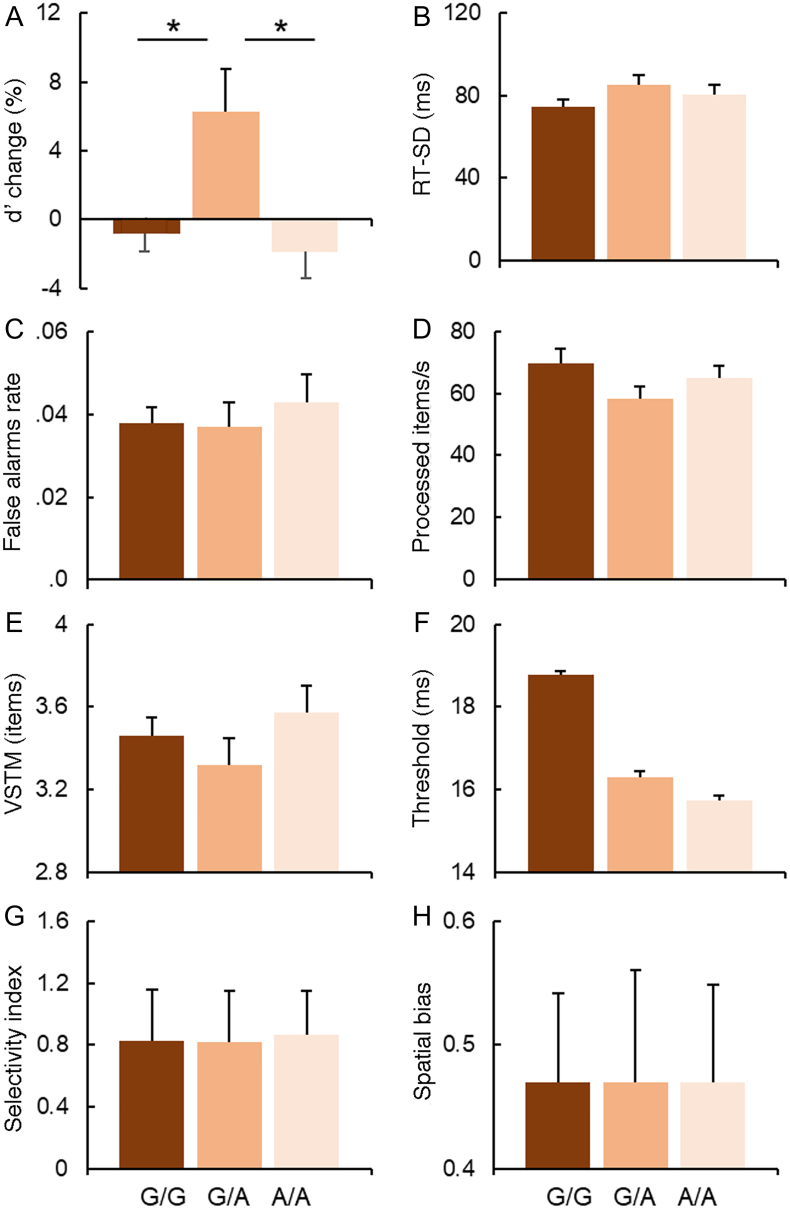
**The effect of DBH 444 G/A genotype status on the different measures of attention functions**: sustained attention **(A)** d’ change and **(B)** RT-SD as assessed by the CPT-SA task performance; **(C)** response inhibition as assessed by the CPT-GNG task performance; **(D)** processing speed**, (E)** VSTM capacity, **(F)** perceptual threshold, **(G)** top-down selectivity and **(H)** spatial bias as estimated based on the CombiTVA task performance. The G/A group showed a striking increase in target sensitivity over time compared to other two groups (*p = .005). No other group differences in attentional function were found with respect to the DBH genotype. Each column chart represents mean task measures ± SE.

**Fig. 5:**
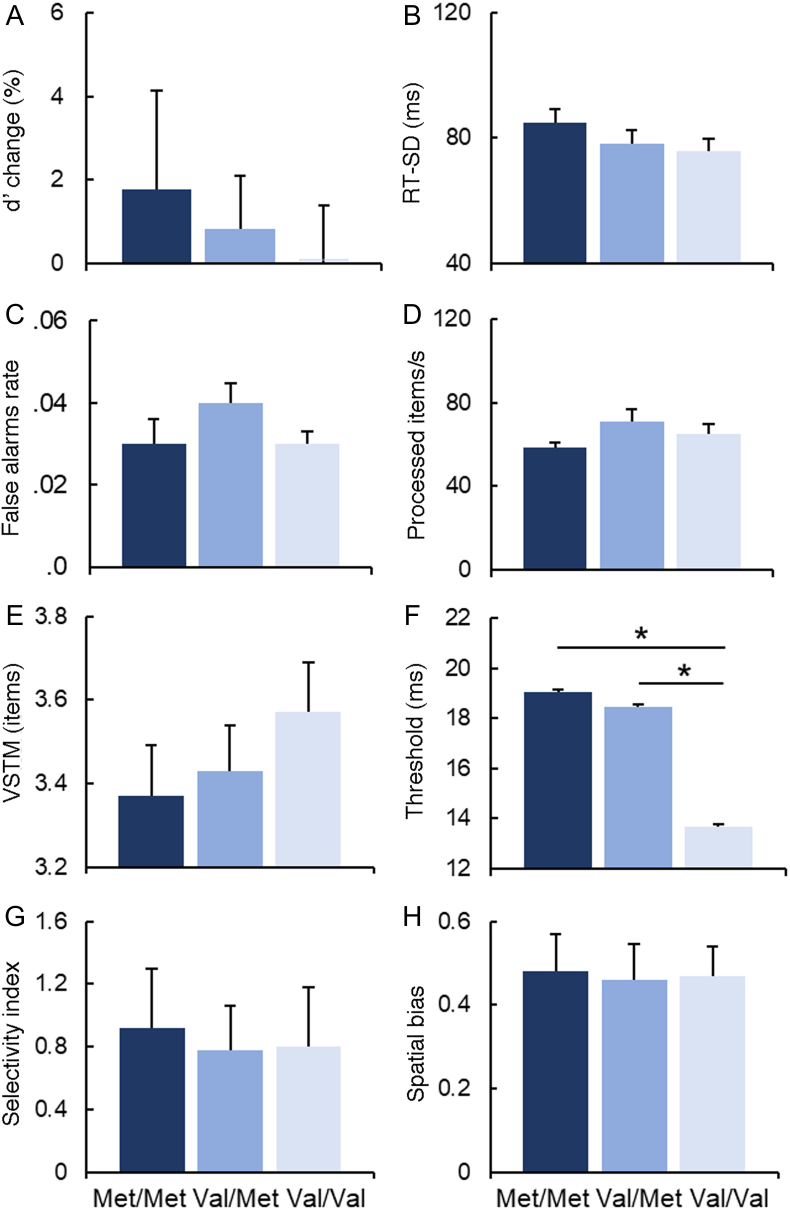
**The effect of COMT Val**^**158**^**Met genotype status on the measures of attention functions**: sustained attention **(A)** d’ change and **(B)** RT-SD as assessed by the CPT-SA task performance; **(C)** response inhibition as assessed by the CPT-GNG task performance; **(D)** processing speed**, (E)** VSTM capacity, **(F)** perceptual threshold, **(G)** top-down selectivity index and **(H)** spatial bias as estimated based on the CombiTVA task performance. The Val/Val group showed a significantly lower perceptual threshold compared to other two groups (*p ≪ .05). No other group differences in attentional function were found with respect to the COMT genotype. Each column chart represents mean task measures ± SE.

As illustrated in Table 1 a stepwise regression analysis showed a significant effect of DBH genotype on the capacity to maintain attention over time (d’-change, sustained attention) as measured by the CPT-SA task performance, and a significant effect of the COMT genotype on the perceptual threshold estimated based on the CombiTVA task performance. In the following sections we systematically report results of the additional analyses conducted to further explore these effects. Fig. 4, Fig. 5 present descriptive data for the distinct measures of attention assessed by the three tasks as a function of either the DBH 444 G/A or the COMT Val^158^Met genotype status, respectively.

**Table 1:** The output from the stepwise regression analysis employed to examine the effect of DBH 444 G/A and COMT Val^158^Met genotype status and their interactions (independent factors) on different aspects of attention (dependent variables). Significant results are reported in bold.

Attention index	Factors	Coefficients	Standard Errors	*p*-values
d’ change (‘sustained attention’)	COMT **DBH** COMT X DBH	0.0236 **0.1164** 0.0104	0.0448 **0.0454** 0.0196	0.59 0.01* 0.59
RT-SD (‘performance stability’)	COMT DBH COMT X DBH	4.4739 5.2376 1.8233	3.2910 3.3418 1.0834	0.17 0.11 0.09
False-alarms rate (‘response inhibition’)	COMT DBH COMT X DBH	0.0016 0.0003 0.0009	0.0039 0.0040 0.0013	0.68 0.93 0.47
Visual short-term memory capacity	COMT DBH COMT X DBH	− 0.1104 − 0.0612 − 0.0377	0.0749 0.0767 0.0247	0.14 0.42 0.13
Perceptual threshold	**COMT** DBH COMT X DBH	**2.7251** − 1.1731 − 0.3907	**0.9936** 1.0100 0.4684	0.007* 0.25 0.41
Processing speed	COMT DBH COMT X DBH	− 1.0397 − 2.3814 − 0.8914	2.0286 2.0559 0.6663	0.60 0.24 0.18
Spatial bias	COMT DBH COMT X DBH	0.0033 − 0.0015 0.0018	0.0086 0.0088 0.0028	0.70 0.86 0.51
Top-down selectivity index	COMT DBH COMT X DBH	0.0600 − 0.0016 − 0.0074	0.0341 0.0349 0.0161	0.08 0.96 0.64

### The effect of DBH 444 G/A genotype status on sustained attention (d’ change)

The regression analysis revealed that the DBH genotype status affects how the performance on CPT-SA task changes over time (d’ change; Table 1). We next conducted a series of complementary analyses to identify the source of the observed effect.

First, we wanted to examine whether the difference in the d’-change index was driven by a significant difference in performance between the two task-halves among the three DBH 444 G/A genotype groups. Thus, we carried out a repeated measures ANOVA, with the task half as a within-subjects factor and, DBH 444 G/A genotype as a between-subjects factor and target sensitivity – d’ (calculated based on CPT-SA task performance) – as the dependant factor. In the above analysis we included COMT Val^158^Met genotype status as a covariate to assure there were no interactions and to co-vary out the effect of other genotype. ANOVA showed a significant interaction of Genotype × Half (F(2,117) = 7.837; p = .001; Partial *η*^2^ = 0.118), There were no other main effects and the COMT genotype did not interact with any of the variables (all p's ≫ 0.1). To validate specificity of the effect of DBH genotype on sustained attention, we conducted a supplementary ANOVA (as above) contrasting the three DBH genotype groups but with respect to the change in perceptual sensitivity d’ between two halves of the CPT-GNG task. The CPT-GNG and CPT-SA tasks have a very similar overall experimental design (same stimulus set and task length) but a different target–distractor ratio. The higher number of targets in CPT-GNG task lowers the requirement for sustained attention, compared to the CPT-SA task. In contrast to the CTG-SA task, no group differences were found with respect to the DBH genotype and d’ measure of change in target sensitivity between two halves of the CPT-GNG task (p = .565).

As illustrated in Fig. 4A, DBH heterozygotes showed a striking increase in performance over time. In contrast, both homozygote groups showed a slight decrease in their performance over time. Post-hoc tests for independent samples demonstrated a significant difference between the performance-change index (d’-change) in the G/A group when compared with the A/A group (t(76) = − 2.926; p = .005; 95% CI[− 0.05;− 0.09]) and with the G/G group (t(81) = − 2.866; p = .005; 95% CI[− 0.42;− 0.07]). The A/A and the G/G groups did not differ from one another (p ≫ .5).

These results survived Bonferroni correction for multiple comparisons (α level, P = .017, corrected for 3 comparisons). Taken together our findings indicate a specific association between the DBH 444 G/A genotype status and sustained attention.

### The effect of COMT Val^158^Met genotype status on perceptual threshold

The regression analysis revealed a significant effect of COMT genotype on perceptual threshold but not on any other measures of selective attention as derived in accordance with the TVA model (Table 1).

As illustrated in Fig. 5F, the perceptual threshold significantly decreased with increased Val allele dosage. A subsequent post-hoc comparison showed a lower perceptual threshold in the Val/Val group compared with both the Val/Met (t(80) = 2.458; *p* = .016; 95% CI[0.91;8.65]) and the Met/Met (t(83) = 2.609; *p* = .011; 95% CI[1.27;9.44]) groups. The Val/Met and the Met/Met groups did not differ (*p* = .79). These results survived Bonferroni correction for multiple comparisons (α level, P = .017, corrected for three comparisons). This trend was also confirmed using a specific test for a linear contrast, with the COMT Val allele dosage as the independent factor. The linear trend contrast was found significant (F(2,122) = 7.516; p = .007).

The perceptual threshold we examined here was defined in accordance with the TVA model (see Kyllingsbaek, 2006) and thus calculated as the minimum exposure duration (i.e., the minimum visual display duration in milliseconds) required to evoke conscious perception. To explore how COMT genotype was related to perceptual threshold, we carried out supplementary analyses focussing on the effect of the COMT genotype on direct measures of behavioural performance, namely on the mean number of errors made and the mean number of identified letters at each exposure duration (10, 20, 50, 80, 140 and 200 ms). Our aim was to examine whether the Val allele dosage indeed affects the overall sensitivity to perceptual signals versus whether it results in different performance strategies.

**Fig. 6:**
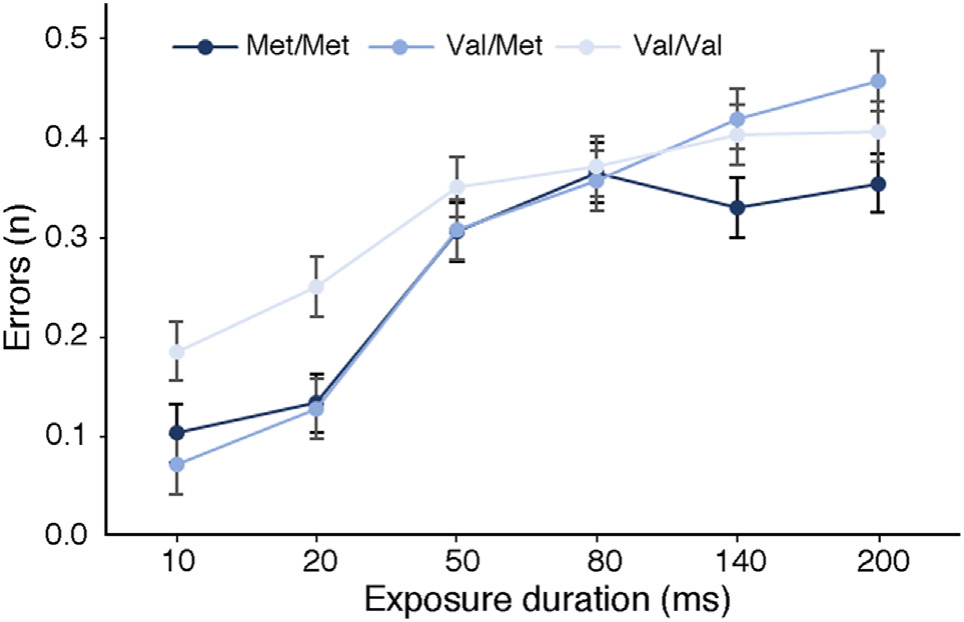
**The association between COMT Val**^**158**^**Met genotype status and the mean number of errors made at different exposure durations.** Data points in each line graph represent mean measures at six different exposure durations ± SE**.**

We first focussed on the effect of COMT genotype on the mean number of errors made at each exposure duration (Fig. 6). A 3 (between subjects factor: COMT genotype) × 6 (within subjects: exposure duration) ANOVA revealed a significant main effect of exposure duration (F(5,610) = 61.246; p ≪ .001; Partial η^2^ = 0.334). There was an interaction between exposure duration and COMT genotype (F(10,610) = 2.202; p = .016; Partial η^2^ = 0.035) but no main effect of COMT genotype (p = .226). Post-hoc comparisons revealed that the interaction was driven by significant differences in the number of errors made between Val/Val genotype compared to Val/Met and Met/Met genotypes at the two shortest exposure durations (10 ms and 20 ms). Specifically, there was a significant difference in the mean number of errors made between the Val/Val group and the Val/Met groups at 10 ms (t(80) = 2.515; *p* = .014;95% CI[0.02;0.20]) and 20 ms (t(80) = 2.335; *p* = .022; 95% CI[0.01;0.22]) exposure durations, as well as between the Val/Val group and the Met/Met group at 20-ms exposure duration (t(83) = 2.151; *p* = .034; 95% CI[0.00;0.22]). In contrast, post-hoc comparisons revealed no significant group differences in the mean number of errors made at longer exposure durations i.e., 50, 80, 140 and 200 ms.

**Fig. 7:**
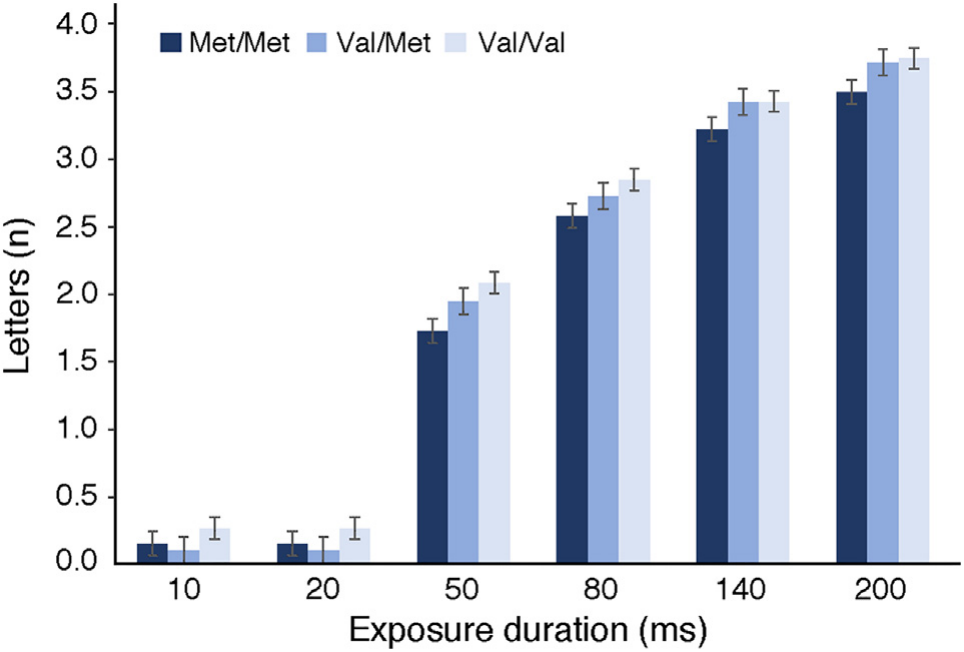
**The association between COMT Val**^**158**^**Met genotype status and the mean number of reported letters at different exposure durations.** Each column represents mean measures for each COMT group at six different exposure durations ± SE.

As illustrated in Fig. 6 and further demonstrated by the first series of comparisons (as above), the Val/Val carriers compared to the two other groups (Val/Met and Met/Met) were the least accurate (made highest number of errors) when comparing the responses made at the two shortest exposure durations i.e., 10 and 20 ms. To further explore the effect of performance strategy on the estimated perceptual threshold, we examined whether at the shortest exposure durations, the Val/Val group not only made more errors but also overall reported more letters irrespective of whether the reports are correct or not (Fig. 7). Thus, we applied a series of post-hoc tests comparing the mean number of reported letters at the different exposure durations between the genotype groups. There was a significantly larger number of reported letters in the Val/Val group compared to the Val/Met group at 10 ms (t(80) = − 2.536; *p* = .013; 95% CI[− 0.29;− 0.03]) and 20 ms (t(80) = − 2.261; *p* = .026; 95% CI[− 0.35;− 0.02]) exposure durations. Furthermore, the Val/Val reported more letters compared to the Met/Met group at 20 ms (t(83) = − 2.297; *p* = .024; 95% CI[− 0.36;− 0.02]) and 50 ms (t(80) = − 2.121; *p* = .037; 95% CI[− 0.68;− 0.02]; please note that at 50 ms there were no group differences in the mean number of reported errors) exposure durations. There were no other differences in the number of reported letters for any of the longer exposure durations. To conclude, at the two shortest exposure durations the Val/Val group significantly differs from the other groups by both displaying and increased number of error made and number of reported letters. As the two shortest visual display durations are the closest to the participants' mean perceptual threshold (16.26. ± 9.40; mean ± SD), our findings suggest that the effect of the Val allele on the perceptual threshold might be a result of the trade-off between the number of reported letters and the number of errors made, rather than increased sensitivity to perceptual signals in the Val/Val group. Please note that the findings indicating the differences between COMT genotype groups with respect to the number of errors made and number of reported letters at different exposure durations did not survive Bonferroni correction for multiple comparisons (α level, P = .001, corrected for 36 comparisons).

## Discussion

Here we show dissociable effects of catecholamines on visual attention, by means of examining the impact of two single-nucleotide polymorphisms in COMT (Val^158^Met) and DBH (444 G/A) on inter-individual variability in performance on behavioural tasks measuring sustained attention, response inhibition and selective attention assessed based on the TVA framework (Bundesen, 1990). Specifically, we demonstrate that effective threshold of visual perception in attentional selection is associated with COMT but not DBH genotype, and conversely sustained attention phenotype is associated with DBH but not COMT genotype.

Prior research indicates robust modulation of the PFC by catecholamines (e.g., Chandler et al., 2014, Clark and Noudoost, 2014), as well as some of the cognitive processes underlying visual attention (e.g., Bellgrove et al., 2007, Greene et al., 2009, Greene et al., 2010, Newman et al., 2012, Newman et al., 2014, Zozulinsky et al., 2014, Schneider et al., 2015). Even though in principle we replicate some of the previous findings linking functional polymorphisms in COMT and DBH to visual attention, in contrast to former reports our study directly demonstrates differential effects of these two polymorphisms on distinct cognitive processes underlying visual attention, rather than simply examines their effects on a singular attentional mechanism. Consequently, our findings provide novel experimental evidence that dopaminergic and noradrenergic genotypes exert dissociative cognitive effects on visual attention functions.

Furthermore, our study strongly supports the previously suggested notion of a non-linear “inverted U-shaped” association between levels of catecholamines and cognitive functioning (Arnsten and Goldman-Rakic, 1998, Arnsten and Li, 2005, Robbins and Arnsten, 2009, Cools and D'Esposito, 2011) by providing a novel genetic evidence that either insufficient or excessive catecholaminergic activity may have detrimental effects on sustained attention.

We present here experimental findings suggesting an association between COMT genotype and one of the selective attention measures, assessed based on the Bundesen's TVA framework (Bundesen, 1990, Kyllingsbaek, 2006). When considering selective attention, the TVA model evaluates factors such as the capacity of the short-term memory supporting visual attention, the minimum exposure time required for visual stimuli to be perceived (perceptual threshold), the speed at which stimuli are processed once perceived, the attentional weights allocated to perceived elements (spatial bias), and the efficiency of top-down control (distractibility). Prior studies found associations between the Val^158^Met COMT polymorphism and cognitive performance indicative of working memory capacity, orientation bias, top-down control and distractibility (e.g., Bertolino et al., 2006, Tan et al., 2007, Holmboe et al., 2010, Zozulinsky et al., 2014, Jaspar et al., 2015, Schneider et al., 2015). Thus, we were somewhat surprised to find the effect of COMT genotype on perceptual threshold, rather than other TVA-derived attentional parameters, in particular visual short term memory, top-down selectivity or attentional weights. It should be also noted that in contrast to the prior studies indicating a cognitive benefit of Met allele (Holmboe et al., 2010, Jaspar et al., 2015, Schneider et al., 2015), our findings point to cognitive benefit of the Val allele, at least in the context of visual attention. Similar inconsistency has been previously reported in meta-analysis examining the cognitive effects of COMT (Val^158^Met) polymorphism, which have recounted various discrepancies and sometimes even opposing effects of the Met versus Val alleles on the performance of diverse cognitive tasks (Mier et al., 2010). However, our findings are also consistent with an alternative explanation. Namely, our secondary analyses indicate that the effect on Val allele dosage on the perceptual threshold (i.e., threshold below which no effective processing of visual stimuli takes place) might be a result of a trade-off between the number of reported letters and the number of errors made rather than directly on overall sensitivity to perceptual signals. At the short visual display durations, the increase in Val allele was associated with both an overall higher number of reported letters and errors made, potentially suggesting that Val allele is associated with pursuing a “high risk strategy” in performing the visual report task. In agreement with this observation Farrell et al. (2012) reported an association between the Met allele and high risk aversion (“low risk strategy”), as demonstrated by performance on a task involving a choice between gambling high versus low monetary amounts.

To assess links between inter-individual differences in sustained attention and catecholaminergic genotypes, we employed previously developed masked version of the continuous performance task (Shalev et al., 2018b). In contrast to a recent study by Park and Waldman (2014), who found an effect of the COMT (Val^158^Met) polymorphism on behavioural measures of sustained attention assessed with the continuous performance task, we did not observe any effects of COMT on any of the sustained attention indices. However, there are several possible explanations for this discrepancy. Firstly, Park and Waldman (2014) studied the influence of COMT (Val^158^Met) genotype status not in healthy participants (our study) but in a clinically selected sample consisting of children with a diagnosis of either ADHD, conduct disorder, or oppositional defiant disorder, and their healthy (not fulfilling the criteria for diagnosis) siblings and twins. This raises the possibility that COMT might be more relevant in clinical population, with sustained attention phenotype being one of the core functional trait of a disease. Secondly, while Park's and Waldman's study examined the association between the COMT (Val^158^Met) polymorphism and sustained attention in children of both genders (mean ± SD age, 12.2. ± 3.2), our study recruited only adult males (mean ± SD age, 44.8. ± 4.43) due to the previously reported sexual dimorphisms in the COMT function (Tunbridge and Harrison, 2011). Thus, the observed discrepancy, perhaps may also result from developmental variance, consistent with changes in COMT expression levels and activity across human lifespan (Tunbridge et al., 2007). Finally, as both the task and the task-derived measures differ somewhat between the two studies, the derived findings may not be directly comparable (please see Shalev et al., 2018b for further discussion on the issue of continuous performance task design and the validity of derived sustained attention measures).

The data presented here suggest an effect of DBH (444 G/A) genotype on sustained attention. We observed here that G allele dosage was associated with the d’-change, indicative of change in target sensitivity over time i.e., indexing sustained attention. However, we have not observed any effects of DBH genotype on either performance fluctuation, as measured by the standard deviation of reaction times, or response inhibition. A similar association between DBH genotype and sustained attention has been reported by Greene and colleagues (Greene et al., 2009), although it should be noted that they showed the link between another common functional DBH polymorphism (− 1021 C/T) and the number of commission errors during performance of a sustained attention to response task (SART). Greene et al. (2009) attributed the genotype differences in attentional performance to changes in noradrenaline (better cognitive performance resulting from increased levels of noradrenaline) based on known effects of noradrenaline on alertness and arousal (for review see Aston-Jones and Cohen, 2005, Thiele and Bellgrove, 2018). This noradrenaline-centric interpretation is consistent with the findings presented here, although an effect of dopamine (or the interaction between catecholamines) on the observed behavioural effects cannot be ruled out. As a caveat, the behavioural phenotype observed in DBH knockout mice (who have a complete inactivation of DBH gene) has been attributed to both a complete lack of noradrenaline, and hypersensitive dopamine signalling (Mitchell et al., 2008). It should be also noted that studies examining the effects of DBH SNPs on decision-making performance and reward related behaviours have interpreted their findings in terms of dopaminergic rather than noradrenergic effects (e.g., Parasuraman et al., 2012).

Both rodent and primate studies provide compelling evidence for the model of an “inverted U-shaped” action of both dopamine and noradrenaline in the prefrontal cortex linked to cognitive abilities such as working memory and executive functions (e.g., Zahrt et al., 1997, Granon et al., 2000, Arnsten, 2007, Vijayraghavan et al., 2007). Similarly, using functional magnetic resonance imaging and pharmacological manipulations in human participants, Gibbs and D'Esposito demonstrated an inverted U-shaped dose effect of dopamine on working memory performance and brain activity (Gibbs and D'Esposito, 2005). Interestingly, in their review of pharmacological studies examining modulatory effects of catecholamines on cognitive performance, Robbins and Arnsten suggested that catecholamines might exert both linear and non-linear effects depending on the brain area under control and the nature of the performed cognitive task (Robbins and Arnsten, 2009). In the current study, we observed a significant cognitive benefit (relatively improved performance) in DBH 444 G/A heterozygotes compared to both A/A and G/G homozygotes, consistent with the hypothesis that both too little and too much catecholamine signalling (presumably here noradrenaline; see comments above) might impair sustained attention. In contrast, our COMT Val158Met findings reflect a linear allele-dose related model with regard to visual attention function, consistent with earlier studies of executive function (Egan et al., 2001, Holmboe et al., 2010, Jaspar et al., 2015, Schneider et al., 2015). Our findings therefore support the existence of a linear relationship between COMT genotype status and selective attention, with increasing Val allele dosage leading to a lower perceptual threshold potentially driven by a high-risk report strategy. Overall our findings are in agreement with Robbins and Arnsten's proposal that the linear versus non-linear effects of catecholamines, dopamine and noradrenaline, vary depending on the brain area and cognitive function modulated by these neurotransmitters, highlighting the importance of high-precision behavioural testing which enable the dissociation of possible genetic effects on distinct attentional processes (Robbins and Arnsten, 2009).

In conclusion, our findings provide novel genetic evidence for (i) dissociative dopaminergic and noradrenergic effects on visual attention; (ii) “inverted U-shaped” catecholaminergic action on human visual attention, specifically that either insufficient or excessive catecholaminergic activity may have equally detrimental effects on sustained attention. Thus, our study supports the notion of dissociative dopaminergic and noradrenergic modulation of the PFC, which exerts control over cognitive processes underlying visual attention, and indicates a need for precise pharmacological targeting of specific cognitive mechanisms in attention disorders.
